# A Mouse Model for Studying Nutritional Programming: Effects of Early Life Exposure to Soy Isoflavones on Bone and Reproductive Health

**DOI:** 10.3390/ijerph13050488

**Published:** 2016-05-11

**Authors:** Wendy E. Ward, Jovana Kaludjerovic, Elsa C. Dinsdale

**Affiliations:** Department of Kinesiology, Brock University, 1812 Sir Isaac Brock Way, St. Catharines, ON L2S 3A1, Canada; jovanakal@gmail.com (J.K.); elsadinsdale@gmail.com (E.C.D.)

**Keywords:** bone mineral density, bone strength, bone structure, development, mice, isoflavones, nutritional programming, reproductive health, soy

## Abstract

Over the past decade, our research group has characterized and used a mouse model to demonstrate that “nutritional programming” of bone development occurs when mice receive soy isoflavones (ISO) during the first days of life. Nutritional programming of bone development can be defined as the ability for diet during early life to set a trajectory for better or compromised bone health at adulthood. We have shown that CD-1 mice exposed to soy ISO during early neonatal life have higher bone mineral density (BMD) and greater trabecular inter-connectivity in long bones and lumbar spine at young adulthood. These skeletal sites also withstand greater forces before fracture. Because the chemical structure of ISO resembles that of 17-β-estradiol and can bind to estrogen receptors in reproductive tissues, it was prudent to expand analyses to include measures of reproductive health. This review highlights aspects of our studies in CD-1 mice to understand the early life programming effects of soy ISO on bone and reproductive health. Preclinical mouse models can provide useful data to help develop and guide the design of studies in human cohorts, which may, depending on findings and considerations of safety, lead to dietary interventions that optimize bone health.

## 1. Introduction

Osteoporosis results in fragility fractures that are associated with substantial morbidity and mortality. In Canada, one in three women and one in five men will experience an osteoporotic fracture during their lifetime, and these estimates are similar for other countries [1,2]. Moreover, bone health in developed countries will continue to worsen as the population ages. Dietary strategies provide an opportunity to reduce the risk of fragility fractures during aging, particularly if introduced during early stages of development when the genome is sensitive to epigenetic programming. 

Over the past decade, our research group has characterized and used a mouse model to demonstrate that “nutritional programming” of bone development occurs when mice receive soy isoflavones (ISO) during the first days of life. This can be defined as the ability for diet during early life to set a trajectory for better or compromised bone health at adulthood. We have shown that CD-1 mice exposed to soy ISO during early neonatal life have higher bone mineral density (BMD) and greater trabecular inter-connectivity in long bones and lumbar spine at young adulthood (4 months of age) [3,4,5,6,7]. Moreover, the higher BMD and improved bone structure allows these skeletal sites to withstand greater forces before fracture [3,4,5,6,7].

Preclinical mouse models, such as the CD-1 mouse model, are not without limitations when extrapolating findings to the human situation. Nonetheless, these models provide useful data that can help develop and guide the design of studies in human cohorts, such as retrospective or prospective studies, which may, depending on findings and considerations of safety, lead to dietary interventions in humans. This review discusses the development of a mouse model to better understand how exposure to novel food components during early life can impact later bone health. While our first studies focused on the phenotype (higher BMD, better bone structure, and greater bone strength), to more fully address questions about this model, we needed to better understand how the dose, route, and frequency of ISO administration affects serum ISO levels of developing mice, as well as how the duration of ISO administration modulated outcomes of bone health. Given that the chemical structure of ISO resembles 17-β-estradiol and binds to estrogen receptors in reproductive tissues, it was prudent to expand analyses and evaluate reproductive health. Thus, studies were performed to assess estrus cycle length, fertility, and histomorphology of reproductive organs [8,9]. This review highlights aspects of our studies that have used the CD-1 mouse model to understand early life programming effects of soy ISO on bone and reproductive health. 

## 2. Experimental Section 

**Figure 1:**
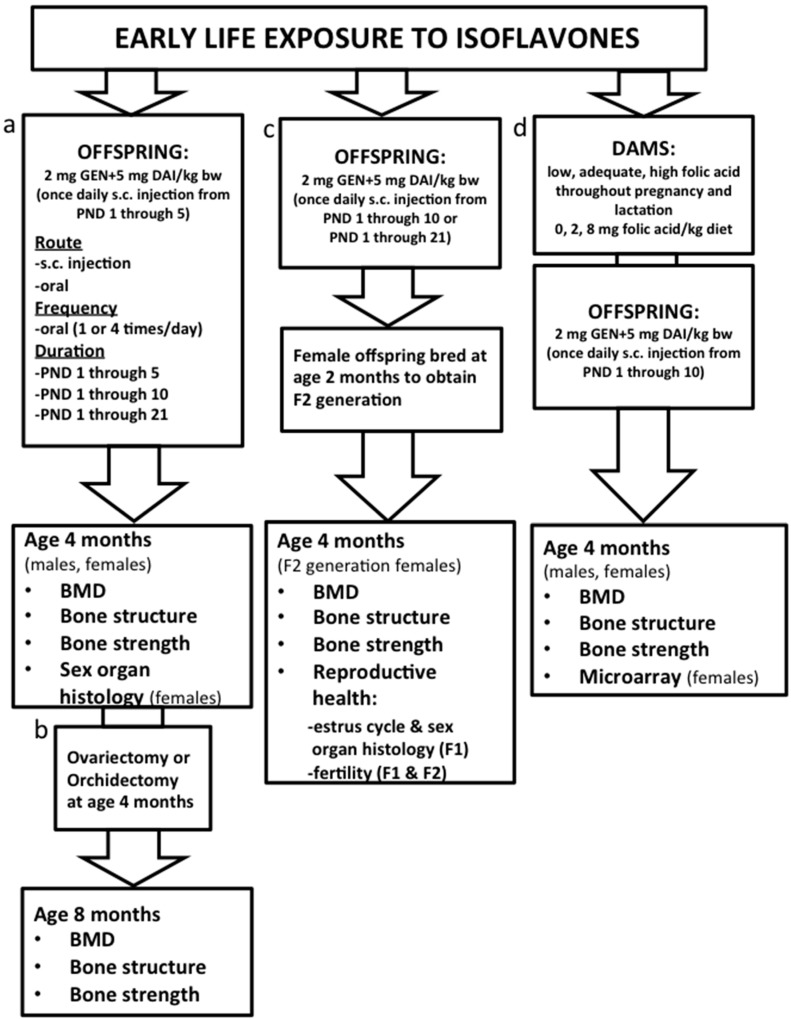
A summary of published studies in which we have used CD-1 mice to provide insight into how exposure to a novel food component such as soy isoflavones (ISO) programs outcomes of bone health. (**a**) Effect of early life exposure to soy ISO on bone health at adulthood; (**b**) Early life exposure to soy ISO and protection against deterioration of bone tissue after ovariectomy or orchidectomy; (**c**) Sustained benefit of early life exposure to soy ISO in subsequent generation: potential transgenerational effect; (**d**) Providing folic acid as a methyl donor to enhance nutritional programming by soy ISO. Effects on reproductive health were assessed within (**a,c**). DAI: daidzein; GEN: genistein; PND: Postnatal day; BMD: Bone mineral density.

Figure 1 is a summary of published studies in which we have used CD-1 mice to provide insight into how exposure to a novel food component such as soy ISO programs health outcomes. While there are many different *in vivo* studies depicted in Figure 1, the studies are shown in four parts (a, b, c, d) and the designs of each are briefly discussed in that order. Further details are available in the primary papers [3,4,5,6,7,8,9,10,11,12]. 

**a. Effect of early life exposure to soy ISO on bone health at adulthood (Figure 1a)** [3,4,7,9,10]. At birth, male and female offspring were randomized to corn oil control (CON); daidzein (DAI); genistein (GEN); or a combination of DAI and GEN from postnatal day (PND) 1 through 5 by subcutaneous injection, once daily. Pups remained with dams during the study and were weaned at PND 21. At weaning, mice were group-housed according to sex and fed AIN93G diet until 4 months of age. The combined dose of DAI + GEN was calculated to present the quantity and ratio of each ISO in soy protein-based infant formula (2 mg DAI/kg body weight; 5 mg GEN/kg body weight; or 2 mg DAI + 5 mg GEN/kg body weight). After determining the positive bone phenotype, we also determined if the route of delivery or frequency of administration altered serum levels of ISO, specifically DAI and GEN. To do this, ISO were administered from PND 1 through 5 by subcutaneous injection *versus* oral administration (both once per day); and oral administration once daily was compared to four times daily. Other studies were performed to determine if the duration affected bone outcomes; the designs were the same as that described for “a”, but with varying lengths of isoflavone administration (from PND 1 through 5; PND 1 through 10; or PND 1 through 21). In females, histology of reproductive organs (ovary, oviduct, uterus, cervix) at 4 months of age was also assessed.

**b. Early life exposure to soy ISO and protection against deterioration of bone tissue after ovariectomy or orchidectomy (Figure 1b)** [11]. After determining that early life exposure to ISO, particularly DAI + GEN, resulted in higher BMD, improved bone structure and greater bone strength, a follow-up study determined if these benefits sustained bone health after ovariectomy (females) or orchidectomy (males). Thus, the study described in “a” was performed; however, at 4 months of age, mice were ovariectomized or orchidectomized and were kept until 8 months of age, at which point bones were collected for analyses. 

**c. Sustained benefit of early life exposure to soy ISO in subsequent generation: potential transgenerational effect (Figure 1c)** [8,12]. Using a similar design as described for “a”, female offspring that had received CON or ISO (2 mg DAI/kg body weight + 5 mg GEN/kg body weight) from PND 1 through 10 or PND 1 through 21 were bred at 2 months of age with males who had not received any ISO to determine if benefits to bone health were transferred to second generation (F2) female offspring. Bone tissue from F2 females was collected at 4 months of age. Within this design we also measured estrus cycling in the female offspring (F1) treated with CON or ISO, fertility and histology of reproductive organs (ovary, oviduct, uterus, cervix). Fertility was also measured in F2 females.

**d. Providing folic acid as a methyl donor to enhance nutritional programming by soy ISO (Figure 1d)** [5,6]. Based on findings in the broader literature, we hypothesized that ISO may be mediating positive effects on bone outcomes through DNA methylation, and therefore designed an experiment to determine if providing a supplemental level of a methyl donor (folic acid) resulted in greater benefits to bone health. The study design was similar to that described in “a”, but included a dietary intervention for dams. Prior to breeding, dams were randomized to low (0 mg folic acid/kg diet); adequate (2 mg folic acid/kg diet) or supplemental (8 mg folic acid/kg diet) folic acid and were maintained on their respective diet throughout pregnancy and lactation. At 6 weeks of age, bone tissue was collected from a subset of female offspring and microarray analysis (Illumina array for RNA analysis) and DNA methyltransferase expression was measured. At 4 months of age, bones were collected for later analyses. 

**BMD, Bone Structure, and Bone Strength (males and females):** At 4 and 8 months of age, BMD, bone structure, and bone strength were measured using dual energy X-ray absorptiometry (pSabre, Orthometrix), a material testing system (Model 442, Instron), and micro computed tomography (Model MS0900325-0010, GE Healthcare System), as previously reported [6]. Femurs and lumbar vertebra (LV) were analyzed. 

**Reproductive Health (females):** Estrus cycle length was measured by evaluating vaginal smears for phase of the cycle. Smears were taken at the same time each morning over a 14-day period. Fertility was assessed by placing one female with a male until a copulation plug was observed. If a plug was not observed after 14 days of housing the male and female together, the male was removed from the cage and a second mating opportunity was provided after a period of one month with a different male. For sex organ histology, tissue sections were stained with H&E and evaluated by light microscopy. The ovary, oviduct, uterus, and cervix were analyzed to determine the presence of abnormalities. These methods are reported in previous papers [8,9].

**Microarray (females):** RNA extracted from long bones of mice exposed to control or ISO enriched diet with or without folic acid was used for microarray analyses [6]. 

## 3. Discussion

### 3.1. Characterization of the Mouse Model: Determining Route and Frequency of Administration of ISO

The choice of animal model depends on the scientific question and the stage of the life cycle being investigated. Practical considerations such as body size, length of time required to reach adulthood, and the form of the intervention (*i.e.*, whole food or purified compound) often dictate the choice of animal model used. While mouse models will always have limitations when extrapolating findings to the human scenario, there have been a number of considerations to ensure that this model is optimized to best represent the potential effects of early life exposure to soy ISO. 

There are a number of reasons why we and other groups have used mice to study the effects of neonatal exposure to soy ISO at adulthood. These reasons include a short gestation period and a rapid rate of development; sexual maturity is reached by 2 months of age [13,14,15,16] and bone mineral density and strength plateau in both male and female CD-1 mice by 3 and 4 months of age [17]. Also, CD-1 mothers have proven to be amenable to cross-fostering of pups at birth to reduce potential litter effects from skewing findings. We consistently experience a very low rate of cannibalism of litters even with human handling to cross-foster and determine the sex of each pup. Also, the CD-1 mouse model has been used extensively to study environmental estrogens (*i.e.*, bisphenol A and diethylstilbesterol (DES), and thus allows for comparisons with effects of ISO [18]. Our studies investigating potential positive effects of soy ISO, a dietary estrogen, on bone development were somewhat based on earlier studies showing that administration of synthetic estrogen (DES) during the first five days of life favourably programmed bone development in female mice [19]. Effects in males had not been reported. In our first study investigating early life exposure to soy ISO, we included a group of male and female offspring that were exposed to DES as a positive control [7]. Interestingly, sex-specific responses were observed. As expected, female mice exposed to DES had significantly higher BMD and peak load at the lumbar spine and femur midpoint at 4 months of age when compared to controls, but male mice receiving DES had markedly reduced BMD and peak load at the lumbar spine [4,20].

We also showed that the dose and duration of exposure, rather than route of administration and frequency of exposure, have more profound effects on ISO response in CD-1 mouse tissue [3,10]. Moreover, we observed that female neonatal mice treated with ISO had higher concentrations of serum GEN and DAI than male neonatal mice, which highlighted that female neonates metabolize ISO more slowly than males [10]. Serum concentrations of *O*-DMA and equol, the two metabolites of DAI, were negligible in 5-day-old male and female CD-1 mice because intestinal bacteria and phase II metabolism needed for conversion of aglycones to secondary metabolites (*i.e.*, equol and *O*-DMA) are underdeveloped during early life. 

Due to the small size of a mouse, particularly during the first week of life, it is not possible to feed a sufficient quantity of soy based infant formula (SBIF) to achieve serum levels of soy ISO that resemble those of human infants fed this formula. This is a limitation of this model [10]. Thus, soy ISO are most often administered in aglycone form via subcutaneous injection. A summary from an NIH Workshop on Designing, Implementing, and Reporting Clinical Studies of Soy Interventions identified that it is extremely important to evaluate blood levels of soy ISO in animal models and ensure that they are comparable to the blood levels observed in human populations consuming soy ISO containing products [21]. In one of our first studies, we showed that serum ISO levels of CD-1 pups receiving 5 mg of GEN and 2 mg of DAI/kg of body weight/day by once daily subcutaneous injection resembled the serum ISO levels of human infants fed SBIF [4,22]. Data from human and rodent studies have shown that at adulthood, most of the ISO compounds detected in circulation are present in the conjugated form with less than 5% of total plasma ISO being present in the aglycone form [23,24]. However, during development, enzymatic activity is low in both human infants and neonatal rodents, so developing organisms likely have a limited capacity to catalyze glucuronidation of ISO or generate secondary metabolites such as equol [10,22,25]. Five-day old CD-1 mice have been shown to have an elevated fraction of aglycones (~30%) in serum [26]. Thus, the bioavailability of the aglycone form of soy ISO might be higher during development and thereby may have more pronounced estrogenic effects in tissues. We also conducted a study in which we determined the differences in oral *versus* subcutaneous administration of soy ISO on circulating levels of GEN and DAI [10]. We also evaluated if oral delivery once a day *versus* 4 times a day, to more closely mimic the shorter duration among feeding periods in human infants, resulted in different circulating levels of GEN and DAI. Route of delivery and timing (once a day oral or via subcutaneous injection or orally every 4 h) resulted in similar serum levels of GEN and DAI and main metabolites (equol and O-DMA) [10]. 

### 3.2. Effects on Bone Health: Dose and Duration of Isoflavones and Transgenerational Effects 

Findings from our group [3,4,5,6,7,11] and others [27] have shown that targeting the early postnatal life provides a window of opportunity for soy ISO to improve bone health. Our studies have shown that exposure to soy ISO (GEN or DAI alone or in combination) for the first 5 days of life results in higher bone mineral, improved bone structure, and stronger bones at young adulthood [4,7], and attenuates deterioration of bone tissue during aging [11]. Male and female mice treated with GEN had higher BMD at the lumbar spine (LV1–LV4) and stronger vertebra, as demonstrated by the significantly higher peak load of LV3 [7]. However, the effects were more pronounced in female than male mice at young adulthood. As a result, female mice were protected against deterioration of bone tissue post-ovariectomy, while male mice were not protected against deterioration of bone that is accelerated by orchidectomy [11]. 

Effects of GEN on bone in females were similar to those induced by DES, while in males GEN and DES had divergent effects [7]. These findings suggested that GEN enhances bone development through an estrogen-dependent mechanism in females but not in males. A follow-up study used a similar experimental design but included DAI and a combination of GEN and DAI at a dose that mimicked the serum soy ISO concentrations of infants fed SBIF [4]. The combination of GEN and DAI did not induce greater benefits to bone than either treatment alone, suggesting that GEN and DAI may be competing for the same estrogen receptors. 

As in the previous study, the ISO-induced effects on bone in female mice were similar to those seen with early life exposure to DES, suggesting that soy ISO have a potential estrogen-like effect on bone development. To our surprise, the most profound effects on deposition of bone mineral were observed with DAI exposure, as it was the only treatment to improve bone mineral content (BMC) and BMD at the femur [4]. Microstructural analyses revealed that although exposure to all ISO interventions resulted in higher trabecular thickness and lower trabecular separation at the lumbar spine, DAI had the most profound effect. Accordingly, the improvements in bone mineral and bone structure among females treated with DAI were translated into stronger vertebrae that were more resistant to compression fracture, while the other ISO groups had intermediate effects [4]. This study demonstrated that 5-day exposure to DAI has the most profound biological effect on bone development. However, because soy-based foods contain a mixture of both DAI and GEN, it was relevant to investigate how the combination of these compounds program bone development [11]. 

Thereafter, we designed studies to investigate if and how the duration of soy ISO may alter programming effects on bone development [28]. Mice suckle for the first 21 days of life, so it could be argued that ISO exposure should take place during suckling to mimic the stage of development in which human infants are fed SBIF. However, unlike human infants, mice reach sexual maturation 3 weeks post weaning—a much shorter duration between neonatal life and sexual maturity—suggesting that perhaps treatment should be introduced somewhere between the first 5 and 21 days of life to more closely mimic infants fed SBIF. Data compiled from our studies showed that the first 5 days of life represent a critical window of development during which soy ISO program the lumbar spine (a site rich in trabecular tissue that has a high surface-to-volume ratio and is very metabolically active [4,7,11]), while exposure for the first 10 days of life is needed to program long bones (*i.e.*, femur) that have more cortical bone. Outcomes of lumbar spine were also improved with exposure during the first 10 days of life.

The 21-day exposure resulted in similar benefits to bone as the 10-day protocol, with no added benefit. Thus, these studies identified the first 10 days of life as a critical stage during which soy ISO improves bone development. A follow-up study investigated if the benefits to bone health in females were transferred to the next generation of females [12]. Female offspring were exposed to the same dose of ISO as in the previous studies for the first 10 days of life. Improved bone structure was observed at the femur neck and lumbar spine [12]. Mechanisms for transgenerational inheritance need to be determined.

### 3.3. Mechanisms and Interaction with Supplemental Folic Acid

A growing body of literature suggests that exposure to nutrients or bioactive food components during development can affect the rate of extra-uterine growth and programming of long-term metabolic outcomes by altering gene expression or endocrine regulation [29]. While we have determined a consistent phenotype, particularly in female mice—higher BMD, improved bone structure, and greater bone strength—the mechanism(s) remain uncertain. However, recent efforts in defining estrogen signaling pathways and modes of epigenetic programming have provided some insight into potential mechanisms of action. Because of their structural similarity to 17-β-estradiol, soy ISO can bind to estrogen receptors and interfere with hormonal signaling and/or the production of enzymes and transcription factors [30,31]. However, it is important to recognize that there are two estrogen receptors and that the distribution of these subtypes are distinct and that specific isoflavones have different affinities for these receptors. These changes can in turn induce irreversible effects on many physiological processes (*i.e.*, growth, metabolism, stress response, behavior, ability to reproduce) if exposure occurs during sensitive stages of development. During development, endocrine factors such as hormones and enzymes can alter epigenetic regulation by increasing histone acetylation, the availability of DNA methyltransferases, nucleosome positioning, or the production of non-coding RNAs, as well as program long-lasting changes in hormone secretion and tissue hormone sensitivity [32]. 

A study conducted in agouti mice showed that dams fed a diet rich in GEN during pregnancy and lactation gave birth to offspring that had hyper-methylated CpG islands in the promoter of the agouti gene, higher prevalence of pseudo-agouti phenotype, and a lower incidence of obesity at 15 months of age compared to wild-type mice [33]. Postnatal exposure to ISO has also been shown to stimulate hyper-methylation of specific repetitive elements that coincide with significant down-regulation of estrogen responsive genes involved in hematopoiesis and bone marrow cell development [34]. Thus, it is possible that skeletal improvements at adulthood, induced by early life exposure to ISO, are in part mediated by reprogramming of DNA methylation patterns in the epigenome. 

DNA methylation is driven by a group of enzymes called DNA methyltransferases that recognize CpG dinucleotides of palindromic sequences and catalyze the transfer of one-carbon group from a methyl donor to the cytosine residue. As such, changes in the expression of DNA methyltransferase or the availability of methyl donors/acceptors can alter DNA methylation patterns. To date, three DNA methyltransferases have been identified [35]. DNA methyltransferase-1 (Dnmt-1) is responsible for maintaining patterns of CpG dinuleotides throughout replication cycles. Dnmt-3a and -3b are *de novo* methyltransferases that set up DNA methylation patterns early in development. In 4-week old rats injected with human neuroblastoma SK-N-SH cells that had been isolated from toddlers, treatment with GEN enhanced the expression of tumor suppressor CD5 and p53 by down-regulating the expression of Dnmt-3b [36]. Suppression of Dnmt-3a has also been observed in the uterine tissue of mice exposed to DES during early life [37]. DNA methyltransferases use S-adenosyl methionine (SAM) as the methyl donor for DNA. In the rate-limiting step of the SAM cycle, methylenetetrahydrofolate reductase (MTHFR) irreversibly reduces a derivative of folic acid (5,10-methylenetetrahydrofolate) to 5-methyltetrahydrofolate. As such, the availability of folic acid has an important role in how DNA methyltransferases modulate methylation patterns. The combined effect of administering folic acid with soy ISO has been demonstrated in a study of pregnant rats [38]. *In utero* exposure to folic acid and soy ISO supported post-neural tube closure to a greater extent than either treatment alone [38]. In line with these findings, we recently reported that early life exposure to adequate folic acid (2 mg/kg) with ISO (7 mg/kg of bw) but not supplemental folic acid (8 mg/kg) with ISO (7 mg/kg of bw), results in higher BMD and greater resistance to fracture at the femur and lumbar spine in male and female CD-1 mice [5,6]. 

Because we previously showed that female mice had more pronounced responses to soy ISO and another environmental estrogen, DES [4,11,20], we used females to study the mechanism by which folic acid and ISO modulate bone development. Exposure to adequate folic acid plus ISO or supplemental folic acid alone equally suppressed Dnmt3a expression in bone. Since Dnmt3a has the potential to reprogram the expression of many genes, microarray analyses were used to identify target genes responsible for skeletal augmentation. The microarray analyses, together with traditional qRT-PCR analyses, showed that female mice exposed to supplemental folic acid plus ISO, adequate folic acid plus ISO, or supplemental folic acid alone had 3% of their genes changed in bone at 6 weeks of age, providing evidence that folic acid and ISO improve bone development through an epigenetic mechanism. Interestingly, neuropeptide Y (NPY) was one of the most highly-expressed genes in long bones of females exposed to adequate folic acid and ISO. The promoter of NPY spans over 246 base pairs and has three transcription factor binding sites (NGF-RE, AP-2, and NGFI-A) that contain CpG islands, suggesting that imprinting of female bone may in part be mediated by NPY. Further research is needed to more fully understand the underlying mechanism(s). 

Aside from disruptions in epigenetic programming, it has been hypothesized that soy ISO can interact with steroid or non-steroid hormone receptors on the cell membrane and cytoplasm to trigger non-genomic signaling cascades that recruit secondary messengers (*i.e.*, nitric oxide, receptor tyrosine kinases, G-protein-coupled receptors and protein kinases) and lead to downstream cytoplasmic or transcriptional events [39,40]. For example, activation of estrogen receptors in the plasma membrane of osteoblasts and osteoclasts can induce rapid signaling pathways that stimulate apoptosis of osteoclasts, inhibit apoptosis of osteoblasts, and in turn promote bone growth [41,42,43,44]. In summary, there are several estrogen receptor-mediated signaling mechanisms through which soy ISOs have the potential to induce biological responses in bone. However, the underlying details of these mechanisms are an area of future study.

### 3.4. Effects on Reproductive Health

While early exposure to soy ISO improves bone development in the CD-1 mouse model, adverse effects on reproductive health had been observed in mouse models prior to our studies investigating this same aspect [13,16,28,45,46]. Reduced fertility, infertility, estrous cyclicity, greater pregnancy loss, higher incidence of benign tumors in reproductive organs and vaginal adenocarcinomas, reduced testicular size and sperm count, higher body weight, and greater incidence of autoimmune complications had been reported [13,16,28,45,46]. Of note was that the doses of soy ISO studied were often higher than those we showed to benefit bone metabolism. Thus, we hypothesized that there would be no effect on reproductive health outcomes using our dose of ISO shown to benefit BMD, bone structure, and bone strength. 

Our hypothesis was shown to be incorrect. Exposure to soy ISO for the first 5, 10, and 21 days of life were shown to have adverse effects on reproductive health in our CD-1 mouse model [8,9]. Specifically, reduced fertility was observed and associated with abnormal estrus cycles, fewer corpora lutea in ovaries, and increased incidence of hyperplasia in the reproductive tract following treatment [8]. Among females that received soy ISO during the first 10 or 21 days of life, pregnancy success rates were 55%–60% compared to 100% for controls, while there were no differences observed in the subsequent generation of females (F2) [8]. Subfertility and infertility in treated females (F1 generation) was likely related to fewer days spent in estrus phase and the overall lower number of total estrus cycles. Moreover, altered morphology of reproductive organs likely compromised fertility. Notably, normal mating behaviors, determined by the presence of copulation plugs, were observed. For mice that did not become pregnant, a second breeding trial with known fertile males was conducted. During the second breeding trial, mice exposed to ISO during the first 10 days of life continued to experience a low fertility rate (37%), while no pregnancies were observed in mice who received ISO throughout the first 21 days of life [8]. While fertility and estrous cycle was not studied in mice exposed to ISO for the first 5 days of life, another study showed that exposure to ISO during the first 5 and 10 days of life exerted similar adverse effects on structural development of ovaries and uteri and resulted in a higher incidence of oviduct hyperplasia, atypia, polyps, and cysts in the uterine tissue structure [9]. While the impact of early life exposure to ISO has yet to be fully elucidated, noteworthy adverse effects on sexual and reproductive organ development, endocrine function, and fertility warrant further study. Moreover, recognizing that extrapolating findings from mouse models to human infants is complicated, these data do provide some basis for further investigation regarding the implications for infants consuming SBIF. 

Whether there are any potential harmful effects of consuming SBIF on reproductive health in human infants is inconclusive. There is an overall paucity of data in this area. Some but not all studies in human infants suggest effects of soy formula consumption on reproductive development, though the long-term consequences of these changes on human health are unclear. These effects include increased vaginal cell maturation at 6 months of age [47] and accelerated breast tissue development at 2 years of age [48]. Another study has shown no differences in reproductive organ volume and structural characteristics at 5 years of age in males and females [49].

Several retrospective studies have demonstrated signs of endocrine disruption in adult populations, particularly among women fed SBIF as infants. One study reported prolonged menstruation and increased discomfort during menstruation among females with no differences in more than 30 other reproductive health outcomes for both men and women [50]. More recently, the Study of Environment, Lifestyle & Fibroids (SELF), an ongoing cohort study of 1696 women, demonstrated that women fed SBIF as infants had more than twice the risk of endometriosis compared to unexposed women, as well as fibroids with a larger diameter [51]. Another investigation showed an increased risk of early menarche [52]. This is also an area for future study.

## 4. Conclusions

In conclusion, when using a mouse model to extrapolate findings regarding nutritional programming of bone development to the human situation, careful consideration of many aspects is critical to understand both the potential strengths and limitations of the model. The studies discussed provide a framework for one approach in which we considered the dose of the food component as well as the route, frequency, and duration of administration—in addition to effects on other aspects of health, including reproductive health.
